# Scaling-up food policies in the Pacific Islands: protocol for policy engagement and mixed methods evaluation of intervention implementation

**DOI:** 10.1186/s12937-022-00761-5

**Published:** 2022-02-02

**Authors:** Jacqui Webster, Gade Waqa, Anne-Marie Thow, Steven Allender, Thomas Lung, Mark Woodward, Kris Rogers, Isimeli Tukana, Ateca Kama, Donald Wilson, Sarah Mounsey, Rebecca Dodd, Erica Reeve, Briar Louise McKenzie, Claire Johnson, Colin Bell

**Affiliations:** 1 George Institute for Global Health, University of New South Wales, 1 King Street, Newtown, Sydney, New South Wales 2046 Australia; 2 Pacific Research Centre for the Prevention of Obesity and Non-Communicable Diseases, Fiji National University, Suva, Fiji; 3 Menzies Centre for Health Policy, The University of Sydney, Sydney, Australia; 4 Global Obesity Centre, School of Health and Social Development, Institute for Health Transformation, Deakin University, Melbourne, Australia; 5 George Institute for Global Health, Oxford University, Oxford, UK; 6 Johns Hopkins University, Baltimore, USA; 7 Ministry of Health and Medical Services, Suva, Fiji

**Keywords:** Pacific health, Food policy, Intervention, Salt, Sugar, Health policy analysis, Dietary surveys, Evaluation

## Abstract

**Background:**

There is a crisis of non-communicable diseases (NCDs) in the Pacific Islands, and poor diets are a major contributor. The COVID-19 pandemic and resulting economic crisis will likely further exacerbate the burden on food systems. Pacific Island leaders have adopted a range of food policies and regulations to improve diets. This includes taxes and regulations on compositional standards for salt and sugar in foods or school food policies. Despite increasing evidence for the effectiveness of such policies globally, there is a lack of local context-specific evidence about how to implement them effectively in the Pacific.

**Methods:**

Our 5-year collaborative project will test the feasibility and effectiveness of policy interventions to reduce salt and sugar consumption in Fiji and Samoa, and examine factors that support sustained implementation. We will engage government agencies and civil society in Fiji and Samoa, to support the design, implementation and monitoring of evidence-informed interventions. Specific objectives are to: (1) conduct policy landscape analysis to understand potential opportunities and challenges to strengthen policies for prevention of diet-related NCDs in Fiji and Samoa; (2) conduct repeat cross sectional surveys to measure dietary intake, food sources and diet-related biomarkers; (3) use Systems Thinking in Community Knowledge Exchange (STICKE) to strengthen implementation of policies to reduce salt and sugar consumption; (4) evaluate the impact, process and cost effectiveness of implementing these policies. Quantitative and qualitative data on outcomes and process will be analysed to assess impact and support scale-up of future interventions.

**Discussion:**

The project will provide new evidence to support policy making, as well as developing a low-cost, high-tech, sustainable, scalable system for monitoring food consumption, the food supply and health-related outcomes.

## Introduction

Non-communicable diseases (NCDs), including cardiovascular diseases, diabetes, cancer, and chronic respiratory diseases, are the leading causes of death in the world, killing more people each year than all other causes combined. NCDs also represent the single largest cause of premature mortality in the Pacific Islands [1]. For small Pacific economies, increasingly impacted by climate change and more recently by the COVID-19 pandemic [2, 3], prevention is a must because the increasing cost of treating NCDs is overwhelming [4]. Pacific leaders recognize this, as demonstrated by the plethora of policies, backed by ministerial and bilateral support. This includes the Pacific Monitoring Alliance for NCD Action (MANA) supported by the World Health Organization, the Pacific Community, Pacific Research Centre for the Prevention of Obesity and Non-Communicable Diseases and the Pacific Island Health Officers’ Association [5], whilst in Tonga funds from taxes are being strategically directed to health promotion through Tonga Health [6].

World Health Organization (WHO) country disease profiles show an increasing prevalence of NCD risk factors, such as hypertension and diabetes, in the Pacific [7, 8]. For example, in Fiji, 34% of deaths are from cardiovascular diseases and 22% from diabetes, and 25% of males and 30% of females live with obesity [7]. Poor diets are a major contributor to these deaths. Around one third of the increase in blood pressure over the last two decades, in Samoa, can be explained by rising obesity levels [9] which in turn can be explained by changes in food availability [10]. An underlying driver of these high rates of diet-related diseases is the transition from diets based on locally-grown foods [11, 12] to diets characterized by imported foods, refined oils, sugar, confectionery, and processed meats [13, 14], resulting in high population intakes of salt and sugar.

Evidence that food policy interventions improve diets is increasing. Systematic reviews show that taxes and subsidies create a consistent positive effect on consumption across a range of taxation levels [15]. Modelling studies have demonstrated that fiscal interventions can reduce cardiovascular risk and diabetes incidence, and are largely cost effective [16–18]. Interventions that promote reformulation of processed food to reduce salt and sugar content have also been shown to reduce cardiovascular disease risk [19, 20]. A recent systematic review of school food environment policies demonstrated a positive impact on dietary behaviours whilst highlighting the need for longer term investigations assessing the impact of these policies on children’s metabolic risk [21].

Globally, the World Health Organization (WHO) has established maximum recommended intake levels for salt and sugar consumption [22, 23], and WHO recommends that countries introduce policies to reduce population intake of salt and both free and added sugars through its Global NCD Action Plan [24]. In line with global recommendations, the Government of Fiji implemented a tax on sugar sweetened beverages (SSBs), reduced taxes on imported fruit and vegetables, and adopted a cross-ministerial school food and nutrition policy. Also, the Government of Samoa agreed new food regulations, including taxes for salt and sugar [25] increased taxes on SSBs [26] and has introduced national School Nutrition Standards (2012).

While both countries have established a range of policies, effective implementation is a challenge [27]. For example, compliance to nutrition standards in educational institutions has hovered around 30–40% in Samoa, despite significant efforts by the Ministry of Health to revise, promote, and monitor them [28]. Similarly, in Fiji, a Technical Advisory Group was set up to establish policies to reduce salt, fat and sugar consumption, but there was no clear process for implementation [29].

Significant barriers potentially stand in the way of effective implementation of these policies [13, 30]. These include inappropriate criteria for setting levels for taxes or reformulation, or lack of established processes for implementation and monitoring/enforcement. There is also very little national context-specific evidence on the effectiveness or cost effectiveness of food policy interventions in Pacific Island countries [31, 32].

To help Pacific countries counter this, we have designed a five-year project that will take a comprehensive, empirical approach to understand and strengthen the policy making processes. The emphasis will be engaging communities and policy makers and implementing, at scale, the best policies to reduce diet-related diseases, particularly hypertension and diabetes in Fiji and Samoa.

## Methods

**Fig. 1:**
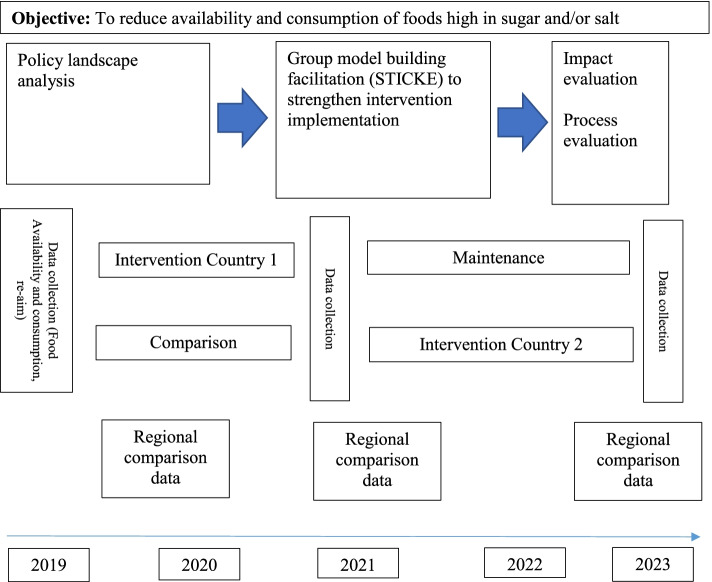
Study design based on type 3 effectiveness-implementation hybrid trial

The overall aim is to test the feasibility and effectiveness, of policy interventions to reduce salt and sugar consumption in Fiji and Samoa, as well as factors that will support sustained implementation of the interventions. We will do this through use of a pragmatic Type 3 implementation effectiveness hybrid trial (See Fig. 1) [33]. We will use an iterative adaptive process to strengthen and monitor food policy interventions, including (1) a policy landscape analysis to map existing policy content, stakeholders and politics; (2) repeated cross sectional surveys to measure dietary intake, food sources and diet-related biomarkers, and implementation of school food policies; (3) Systems Thinking in Community Knowledge Exchange (STICKE) to engage stakeholders to identify, implement and monitor actions to strengthen food policy interventions; (4) evaluation of impact, process and cost-effectiveness of interventions.

The interventions will be staggered so that one country can provide comparison data during the first year, and we will use publicly available data (e.g. WHO Stepwise risk factor (STEPS) survey and the Household Income Expenditure Survey (HIES), as well as data from other national cross-sectional surveys, to understand policy impact.

Main outcome measures: changes in population intake levels of salt and sugar, salt and sugar levels in foods, and food prices (measured through surveys done at baseline and follow-up). Changes in blood glucose and blood pressure and overweight/obesity (measured in consecutive STEPS surveys).

Main process measures: reach, adoption, implementation (context, costs and adaptations made during delivery) of the implemented food policies.

### Policy landscape analysis

The aim of the policy landscape analysis is to identify opportunities for strengthening implementation of food policies to reduce NCDs in Fiji and Samoa. The primary question we will be addressing through the prospective policy analysis is: *What are the potential opportunities and challenges to strengthen policies for prevention of diet-related NCDs in Samoa and Fiji?* Because of their focus on policy change, Walt and Gilson’s health policy analysis triangle [34] and Kingdon’s theory of agenda setting and policy change [35] will inform the study design and development of instruments for data collection. The data collection will focus on 1) the existing policy content, 2) relevant stakeholder actions and perspectives, 3) industry corporate political activity, and 4) the political economy relevant to food and nutrition, informed by previous approaches to policy landscape analysis [36].

#### Documentary analysis

We will conduct a desk-based review of policy content for Fiji and Samoa relevant to diet-related NCD prevention. Policy documents will be identified, based on searches of government websites and direct approaches to government ministries with responsibilities related to food and/or nutrition. Policies in scope will include whole-of-government policy statements, such as the National Development Plan; sectoral policy documents, such as the National Nutrition Policy, and implementation-focussed documents. Relevant content from each of these policies will be extracted to a predetermined matrix based on the study frameworks [36]. This will include information regarding: policy objectives and activities (with reference to the Food Policy Options agreed in the WHO Global Plan of Action for the Prevention and Control of NCDs 2013–2020 [37]); framing of policy problems and solutions (including any mention of gender disparities, institutional responsibilities, co-ordination and implementation mechanisms, and resourcing).

We will draw on this policy content analysis to then conduct a stakeholder analysis to identify the influential actors relevant to diet-related NCD prevention policy, including industry, government and non-government stakeholders [36]. Stakeholders will be defined as: “actors who have an interest in the issue under consideration, who are affected by the issue or who – because of their position – have or could have an active, or passive, influence on decision making and implementation. They can include individuals, organizations, different individuals within an organization, and networks of individuals and/or organizations, i.e. alliance groups” [38]. We will identify such stakeholders and their interests and influence in Fiji and Samoa through 1) searching websites of relevant Government sectors, organizations, institutions and industry actors operating in the country, 2) searches of academic literature, 3) the policy content analysis, and 4) key informant interviews (see details below). We will use a matrix to document the following attributes of identified stakeholders: interest relevant to food and nutrition as a policy issue; level of interest; perceived level of influence/power; source of power/influence; outcomes including impacts on policy [38].

The stakeholder analysis was complemented by an analysis of corporate political activity of the food industry in Fiji and Samoa, based on methods developed by Mialon and colleagues [39]. This will update the analysis already undertaken in Fiji [40]. Data will be collected through searches of industry websites, online media websites and industry analytical reports (e.g. Euromonitor). For each industry actor identified, we will document available information on: Information and messaging; Financial incentives; Constituency building; Legal; Policy substitution; Opposition, fragmentation and destabilization.

#### Key-informant interviews

We will conduct semi-structured interviews with approximately 15–20 key informants in each country, designed to understand the policy and political context, implementation issues, as well as identify facilitators and barriers to implementing different policies. Potential interviewees will be identified based on the desk-based policy content and stakeholder analysis, as well as direct requests to government ministries and other actors relevant to food and/or nutrition policy. The semi-structured interview schedule will be based on the policy theory underpinning the study and will be piloted with two relevant experts who won’t be involved in the final interviews. Interviews will be recorded, where permission is granted, and transcribed in full.

#### Policy analysis

Our analysis will focus on identifying potential policy gaps and opportunities, as well as contextual and politico-economic considerations that might inhibit or foster policy change. The data will be analysed iteratively throughout the data collection process, to identify additional interviewees and potential opportunities to follow up on. We will use NVivo for data management and coding. The data from the policy content, stakeholder and corporate political activity analysis will first be analysed separately, to identify policy strengths and gaps, influential stakeholders, and industry interests and influence. The qualitative data from the interviews will be coded using pre-determined themes based on the study frameworks and then data from all sources will be analysed thematically to 1) understand opportunities for policy change to support improved food systems and nutrition for the prevention of diet-related NCDs, and 2) understand the political economy of food policy in the study countries drawing on Campbell’s approach to political economy analysis [41], which highlights the influence of ideas and understandings of the policy problem and policy solutions, as well as the existing policy and political context (including how long the policy has been in place and when it was evaluated) in shaping opportunities for policy change.

### Repeat cross-sectional surveys to understand dietary intake and behaviours and composition and prices of foods

#### Collating existing relevant data

For each country, we will access and analyse relevant data from existing recent surveys as well as undertake new surveys to inform intervention implementation and monitoring building on the investigators’ recent systematic review [42]. Existing surveys will include the most recent Food and Agriculture Organization (FAO) Household Income and Expenditure Surveys (HIES) [43], WHO Stepwise Approach to Assessment of NCD Risk Factors and (STEPS) [24] and National Food and Nutrition Surveys (NFNS) [44] and any relevant research studies that have looked at diet or food composition. Both Fiji (2010, 2016) [45] and Samoa (2011, 2017) [46] have participated in the Global School Based Student Health Survey, which includes information on body mass index and frequency of soft drink consumption from 12 to 17 year olds. Relevant existing data will be extracted from all recent surveys and tabulated with dates of data collection.

#### 24-h diet surveys

The objective of the 24-h diet recall surveys is to provide an in-depth robust assessment of what adults are eating, including the contributions of different foods to salt and sugar in the diet. 24 h diet recall surveys can be used to accurately assess changes in population intake of nutrients [47]. Urinary analysis is the best way of estimating sodium intake. However, both Fiji and Samoa monitored sodium intake using urinary analysis in 2013 and 2016 [48, 49] and it is not expected to have changed since then. Additionally, spot urine samples will be collected as part of the next WHO STEPS surveys in each country (planned for 2021), so it is not necessary to include urinary sodium analyses as part of this project. In contrast, whilst Fiji has a National Diet and Nutrition Survey (NDNS) from 2015, this will not be repeated until 2025. We will, therefore, undertake dietary surveys in years 1 and 4 in Fiji and 2 and 5 in Samoa.

##### Sampling

Surveys will be undertaken in 3600 adults in Fiji (Viti Levu) in year 1 and 4, and in Samoa (Upolu) in years 2 and 5. We have selected the main islands based on prohibitive cost and time associated with travelling to the outer islands. The samples will be stratified into equal numbers of men and women in two age groups (18–44 and 45–69 years). The sampling strategy will be based on the recent National Nutrition survey (NNS) in Fiji and the most recent WHO STEPS project in Samoa [50]. Each used a two-stage sampling process, based on census information, to obtain representative samples for selected regions. Sampling in Fiji will also be stratified by the two main ethnic groups (Indigenous Fijians, and Indo Fijians). The age, sex and region of randomly selected individuals that do not wish to participate will be recorded, such that response rates and the likely impact of non-response can be estimated.

##### Sample size and power for evaluating impact of food policies

To determine the sample size for salt, we have used the standard deviation from recent surveys in Fiji and Samoa (SD: 3.6) which used urine collection [51], with the knowledge that the standard deviation is likely to be lower using a dietary questionnaire [52], which would mean our sample size calculation will be conservative We have estimated the standard deviation of the proportion of dietary energy from free sugar (SD: 5.4) from a recent large survey (the 2011 Australian Health Survey). With a design effect of 1.5 to account for clustering and stratification, we will need 300 participants in each country at each time-point to reach 0.9 power with a t-test to detect a 1.2 g reduction in salt consumption. For free sugar we estimated that we have 0.9 power to detect a change of 1.8% absolute decrease in the proportion of dietary energy from free sugar between time points. A response rate of about 50% is anticipated, based on our previous work in these settings [53], so 600 individuals will be approached in each country.

##### Recruitment process

For the general population, in line with local customs, individuals will be informed about the study and invited to participate through a face-to-face invitation made by an interviewer visiting the household. Prior to this, contact will be made with the local village chief to request permission to enter the village and carry out the work. Activities to raise community awareness will also be undertaken. If no one suitable is available in the household when the interviewer calls, a repeat visit will be made, or interviewers will attempt to locate the head of the household by visiting local community centres and other meeting places.

##### Data collection process

Research assistants will be trained in the 24-h diet recall survey data collection procedures. The entire enrolment and data collection process will be done face-to-face in English (which is spoken by more than 90% of the populations in these countries) by trained research assistants. Research assistants will be able to assist participants by orally translating written material into the local language. Participant information sheets and consent forms will be available in Hindi and Fijian (for Fiji). Each consented participant will then be assigned a unique ID number. Participants will then be interviewed by the research assistant to:

i. Complete a general questionnaire on basic demographics, disease history and medication use
ii. Complete the 24-h diet recall survey (see below for more details)
iii. Record height, weight, waist circumference and blood pressure [53]

##### Survey procedures

The dietary survey will be a 24 h recall using the multiple pass method [54]. Trained research assistants will use supportive visual tools showing portion sizes for locally-appropriate foods to improve accuracy. Pilot testing of this approach will be undertaken by the research assistants. Discretionary salt intake will also be estimated using previously tested methods [55]. The dietary survey data will be entered into FoodWorks nutrition analysis software [56] using established standard procedures. The FoodWorks program has already been adapted to contain Pacific Island food composition data. The parallel food composition (FoodSwitch [57]) surveys in the two sites (see 2.3 below) will provide more up to date nutrient content data processed foods and these will be incorporated into the FoodWorks dataset. The FoodWorks software enables both an overall estimate of salt and sugar intake as well as the main sources of salt and sugar in the diet.

The *primary outcomes* for the dietary surveys will be gender-disaggregated average levels of sodium and sugar intake levels for adults, and any changes during the study. Key secondary outcomes will be the main sources of sodium and sugar in the diet. Population estimates will be obtained by weighting according to the sample design and the age/gender population structure based on Census data. The results will also be reported for major participant subgroups defined by sex, age and ethnicity (Fiji only) and evidence for differences between subgroups examined using t-tests.

*Reporting results to participants* - The summary results of the 24-h diet recall survey will be distributed to all participants who expressed interest in receiving the results by either email or postal address as part of the consent process. The information provided will include estimated population daily sodium and sugar intakes and how this compares to current national guidelines for salt and sugar. This will be accompanied by simple advice about how to eat a healthy diet low in salt and sugar and high in fruit and vegetables. Phone and email support will be available for 12 months after the last of the baseline and follow-up results letters have been dispatched.

#### Assessing food composition and price of processed foods

The objective of this survey is to measure and assess changes in salt and sugar levels in foods. For this, a standardised approach and a smartphone application will be used to collect data. FoodSwitch is a smart phone application originally developed in Australia and since launched in 7 other countries including the United Kingdom, China and India [57]. The FoodSwitch App can be used to collect food composition data based on back of pack Nutrient Information Panel, which is uploaded into a comprehensive database of nutrition composition. Salt and sugar levels and prices of processed foods in the market will be assessed in years 1 and 4 (2 and 5 for Samoa) at a similar time to the dietary surveys. Data will be collected from the shops that collectively provide the majority (more than 80%) of the processed foods available for purchase in Fiji and Samoa, using the same sampling strategy each year. Food products will be categorised using the existing FoodSwitch food categorisation system. The data fields collected for each product will be brand/manufacturer, product name, food category, serving size, sodium, total fat, saturated fat, and sugar per 100 g. The FoodSwitch App will be used to take photos of the front and back of the pack. Data are then extracted from pictures of the Nutrient Information Panel on the back of packs and inputted by data assistants. Brand/manufacturer name will be recorded in order to establish the proportion of packaged products that are imported compared to the proportion that are produced locally. Price data will be recorded separately. Lists of ingredients will also be recorded.

##### Data analysis and sample size

Mean levels of salt and sugar, overall and by product category, and individual product prices and mean prices for each product category for year 1, 3 and 5 will be calculated. Based on the 2013 processed food composition database, it is expected that data on around 1200 products will be collected each year. We will estimate the paired change in salt and sugar levels between baseline data and each of the follow-up points. Even if the percentage change in composition is highly dispersed (e.g. SD = 20) we should have 0.9 power to detect a 1% change in composition across all categories, or a 10% change for food categories with 50 items. We will analyse the change over time using linear mixed models which account for the repeated measurements.

#### Assessing implementation and compliance with school food policies

Both Fiji and Samoa undertake regular surveys of the school food environment and compliance with healthy school food policies. In Samoa, data is collected on food availability and the food environment within each school at least annually and reported through a multisectoral coordination mechanism [30]. In Fiji, the Ministry of Education, Cultural Heritage and Arts is responsible for implementation and monitoring the Policy on School and Food Canteen. Despite these measures, compliance to healthy food policies has not improved significantly over time. We will analyse these data to generate a baseline report of compliance to standards for Samoa and Fiji. We will also review the policies to understand the degree to which they are likely to be reducing the promotion and availability of foods high in salt and sugar

### Systems thinking in community knowledge exchange (STICKE)

Building on the previous work done in Fiji [58], and informed by baseline outcome data and the policy landscape analysis, we will invite key government, industry and civil society leaders to participate in a structured, facilitated process [59] to build consensus on population level actions to reduce sugar and salt. We will engage 15–20 participants in each country, recruited through known contacts of the existing project team, in a series of deliberative consensus building forums. The objectives will be to understand diets and the drivers of poor diet, map existing interventions, and then discuss barriers and opportunities to strengthening policy implementation.

We will use techniques adapted from community based participatory system dynamics, notably group model-building and software (STICKE), to engage stakeholders in building a causal loop diagram reflecting the complexity of the drivers of unhealthy food availability and consumption and identify new or adapting existing interventions to reduce salt and sugar. Specific food policy interventions identified through a preliminary stakeholder meeting in January 2020 include:

- Taxes on foods or drinks high in salt or sugar – we will identify steps that need to be taken to strengthen existing implementation, including amending the level or type of taxation or extending to other foods.
- Programs to influence the nutrient content of available processed foods - we will identify new policies or opportunities to strengthen implementation of existing policies including engaging industry on reformulation or by working with importing agencies to change the balance of imports (i.e. having nutrient content restrictions to specify which foods that can be imported).
- School food policies and programs - we will review existing programs and identify opportunities to strengthen implementation and broaden reach including through training and capacity building and the strengthening of monitoring and accountability mechanisms.

On completion of the policy landscape analysis, government, industry and civil society leaders will convene every 6 months. The logic model represented by the causal loop diagram built using STICKE will be used to; (i) inform selection of interventions and discuss additional survey data required for monitoring, (ii) use quantitative and qualitative evidence to strengthen intervention implementation, (iii) review interim impact, based on regular monitoring data, and (iv) review the process of implementation to inform feasibility of scale-up, including through recommendations to strengthen monitoring.

**Fig. 2:**
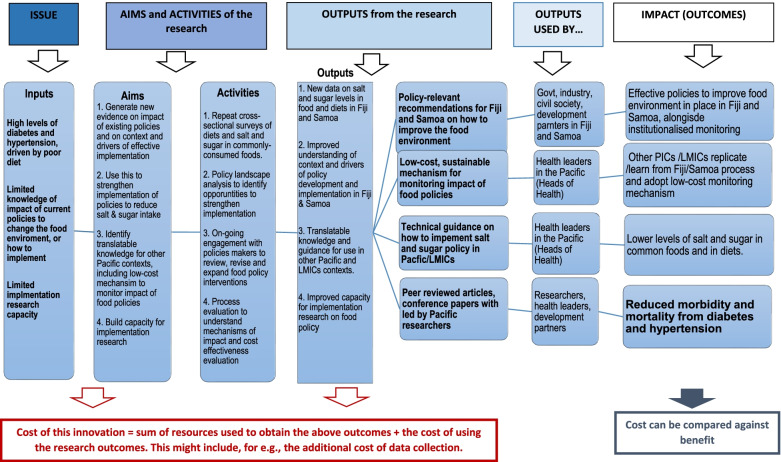
Logic model to identify aims and activities, outputs and impact

A provisional logic model outlining the intended outputs and outcomes of the interventions is provided in Fig. 2.

### Evaluation of impact, process and cost effectiveness

#### Impact evaluation

Depending on the interventions implemented in each country, overall evaluation of impact will examine the extent to which the intervention has had an effect in terms of dietary intake, changes in knowledge, attitudes and behaviours relating to food or the food supply, using the baseline and monitoring surveys in each country. Where school food interventions have been supported as part of the project, additional outcomes, in terms of numbers of schools implementing the interventions, will also be measured. Changes in blood pressure or blood glucose since implementation of the interventions will be extracted from other surveys conducted in Fiji and Samoa throughout the project and the extent to which these are likely to be due to the intervention will be assessed through the process evaluation.

#### Process evaluation

The process evaluation will understand the mechanisms of adoption (factors that led to the decision to implement the intervention) implementation context, and mechanism of impact (including looking at the level of implementation and how this relates to impact) and the feasibility of scaling up (maintenance). Data will be collected throughout the project, building on, and running concurrently with, the policy landscape analysis and STICKE process. Additionally, these data will be used to inform strengthening and adaptations of the interventions in Fiji and Samoa and provide insights into intervention impact and inform feasibility and strategies for scaling up future high-impact policy interventions.

Qualitative data on policy processes will be collected through key informant interviews with food industry, government and consumer actors, schools and education bodies. 15–20 interviews will be undertaken in years 3 and 4 in each country. The qualitative data will be analysed using NVIVO software, with thematic analysis focussed on the barriers and facilitators to the implementation of the intervention and what might need to be done if the program is to be carried out on a wider scale. This approach builds on the UK MRC guidelines for process evaluation which have already been used in Fiji and Samoa [29].

#### Cost effectiveness modelling and evaluation

Country-specific modelling of economic impact will be conducted in Fiji and Samoa. Effect sizes will be estimated through measured changes in consumption from the results of previous studies. Corresponding reductions in the prevalence of NCDs and corresponding mortality will be derived from the academic literature. Costs will be obtained from local stakeholders and government reports to inform the cost-effectiveness analysis. Health outcomes will be estimated in terms of disability-adjusted life years (DALYs) by extrapolating NCD events and survival associated with reductions in salt and sugar consumption. Cost effectiveness evaluation of the implemented policies will be understaken at the end of the program.

### Local training and research capacity building

Because of the growing burden of diet related disease and the related need for expertise in this area, increasing food policy research capacity in both Australia and the Pacific Islands will be an underpinning objective of this project. We will do this based on the principles of the Collective Action Framework which emphasizes “the importance of having a common agenda, common progress measures, mutually reinforcing activities, continuous communication, a backbone organization with staff and a specific set of skills” [60]. The investigator team comprises of senior academics who will provide mentoring and support to the early career researchers in the different countries. The objective will be to increase research capacity at all levels in Australia and the Pacific with a view to translating this experience globally. Effective food policy is needed in Australia as much as it is in Fiji or Samoa and we believe expertise gained through this research can and should be applied in all countries. All phases of the project will be implemented in Fiji and Samoa by a local researcher supported by the Head of the Pacific Research Centre for the Prevention of Obesity and NCDs based at Fiji National University (FNU) in Fiji. In addition, 3–4 research assistants will be recruited during the 4 months required to undertake the surveys during baseline and follow-up assessment. A 5-day program of training in dietary assessment and STICKE will be carried out prior to the initial baseline assessment. Further on-going training and support will be provided during the intervention stage.

## Discussion

Despite increasing evidence of the potential impact of food policy interventions globally [15, 19, 21, 61–63], there is little locally specific evidence and even less evidence about implementation processes for the Pacific. This project will strengthen the scaling up of interventions to improve diet in Fiji and Samoa. Through the use of innovative methods to engage communities and policy makers in understanding policy context and influencers [34, 35], the project will facilitate improved policy processes leading to more effectively implemented policies. The Type 3 implementation effectiveness trial [33, 64], will evaluate the impact of new policies to reduce salt and sugar, at the same time as providing new evidence on the processes required to effectively establish and implement these policies. This is particularly important in Low-Middle Income Countries, where lack of resources and competing priorities create additional challenges for policy implementation [65]. However, it is also relevant in Australia, and other developed countries, where lack of multi-sectoral co-ordination and community involvement and conflicting priorities create challenges. Never has evidence for this been stronger than in the current COVID-19 crisis where health, for once, has come out on top in ongoing battles between public health and the economy but which has nevertheless resulted in extreme strains on already fragile food systems [66]. The adaptive nature of this project means it will be able to respond to this new situation. The importance of the strengthening food policies has never been stronger. In addition, this project will also provide a robust assessment of food consumption patterns and the impact of strengthened implementation of food policies, as well as establishing innovative sustainable systems for monitoring change.

### Strengths and limitations

The strength of this project is that it explores the challenges of implementing food policies in real world settings, with parallel interventions in Fiji and Samoa allowing for shared learning and comparative analyses. The in-depth policy landscape analysis and systems thinking approach to engaging the community and policy makers will hugely increase the chances of culturally appropriate policies being adopted and implemented effectively. The adaptive nature of the methodology means that it is ideally suited to responding to contextual changes affecting implementation, including the additional food security challenges posed by the unfolding COVID-19 situation. Whilst some of the policy analysis is already underway, and staff are being recruited to work with in-country project leads at Fiji National University, it will not be possible to commence the community dietary surveys, which rely on community health workers and include collection of blood samples, until social distancing measures in Fiji have been eased. However, planning, protocol development and training are underway, and with the COVID-19 situation currently seeming relatively stable in Fiji and Australia, we expect that the project should be able to progress with minimum delay.

A further limitation is that, based on practicality and limited resources, the community surveys will be limited to the main island in each country and we will not be able to measure impact in outer islands. However, we expect the research to benefit residents of outer islands because the strategies we co-design with policy makers are targeted at the national level.

Whilst the interventions are context specific, we predict that the evidence generated on strengthening implementation could be used to inform future policy interventions, not only in Fiji and Samoa, but also in Australia and potentially in other small island states. The main limitation is that it is not possible to do a randomised controlled trial and that the interventions are context-specific so the intervention effectiveness findings may not be generalisable to other countries or regions. However, it is likely that the lessons learned can be used to strengthen implementation and monitoring of polices in other countries. In addition, the processes for engaging policy makers and the innovative methods for monitoring may be replicated elsewhere.

### Significance

Diet-related NCDs are one of the biggest contributors to the increasing burden of chronic disease in the Pacific Islands [4]. Without urgent policy action to address diets, chronic disease is predicted to get worse. The emerging COVID-19 pandemic will likely put further strains on food security highlighting the importance of engaging communities in food systems transformation [3]. This project will help contribute to this transformation by providing new evidence about which food policy interventions are most feasible and the factors that lead to effective implementation. Specific outcomes will include a new low-cost system for providing nutrition data for informing and monitoring food policy; evidence of effectiveness of interventions to reduce salt and sugar; information about how to effectively implement such interventions to facilitate scale-up and increased research capacity in the Pacific Islands. Whilst the proposed project will take place in Fiji and Samoa, capacity will also be built in Australia and the results should be applicable to many of the other 20 Pacific Island countries which are the global epicentre of the NCD crisis. Collaboration with government Ministries, involvement of the three multi-lateral agencies and dissemination of findings through the MANA platform [67] will help ensure immediate and effective dissemination of results to support scale-up in Pacific Island countries. The findings will be communicated widely through knowledge translation approaches to maximise public health outcomes.

## Data Availability

Not applicable.

## Abbreviations

DALYs: Disability-adjusted life years
FAO: Food and Agriculture Organization
HIES: Household income and expenditure surveys
MANA: Monitoring alliance for NCD action
NCDs: Non-communicable diseases
NDNS: National diet and nutrition survey
SSBs: Sugar sweetened beverage
SROI: Social return on investment
STEPs: Stepwise Approach to Assessment of NCD Risk Factors
STICKE: Systems Thinking in Community Knowledge Exchange
WHO: World Health Organization
